# Dietary selenium intake and the risk of kidney stones in adults, an analysis of 2007–2018 National Health and Nutrition Examination Survey, a cross-sectional study

**DOI:** 10.3389/fnut.2022.877917

**Published:** 2022-07-22

**Authors:** Minghui Liu, Zhongxiao Cui, Jinbo Chen, Meng Gao, Zewu Zhu, Hequn Chen

**Affiliations:** ^1^Department of Urology, Xiangya Hospital, Central South University, Changsha, China; ^2^National Clinical Research Center for Geriatric Disorders, Xiangya Hospital, Central South University, Changsha, China

**Keywords:** dietary selenium, kidney stones, National Health and Nutrition Examination Survey, calcium oxalate, Randall plaques

## Abstract

**Purpose:**

To evaluate the association between dietary selenium intake and the risk of kidney stones in adults.

**Materials and methods:**

We performed a cross-sectional analysis using data from 2007 to 2018 National Health and Nutrition Examination Survey (NHANES). Dietary intake information of 30,184 participants was obtained using first 24-h dietary recall interview, and kidney stones were presented by a standard questionnaire. The quartile analysis, stratified analysis and non-linearity analysis were used to estimate the association between dietary selenium intake and kidney stones after an adjustment for potential confounders.

**Results:**

The multiple logistic regression indicated that the fourth quantile (Q4) of dietary selenium intake had a lower risk of kidney stones than the first quantile (Q1) in Model 3 (OR 0.82, *P* < 0.05). The stratified analyses indicated there were statistical differences between dietary selenium intake and kidney stones among younger (age < 50) (OR 0.65, *P* < 0.01), male (OR 0.73, *P* < 0.01) and overweight/obese (BMI ≥ 25.0) (OR 0.80, *P* < 0.05) individuals in Model 3. The non-linear relationship was founded between dietary selenium intake and kidney stones in all participants, younger, male and overweight/obese individuals after adjusting for confounding factors.

**Conclusion:**

Our study revealed an inverse relation between the level of dietary selenium intake and the risk of kidney stones for the United States population, especially for younger (age < 50), male and overweight/obese (BMI ≥ 25.0) individuals. The study provides preliminary guidance on dietary selenium intake for the prevention of kidney stones in different populations. Further studies are required to confirm our findings and clarified the biological mechanisms.

## Introduction

Nephrolithiasis is a common disease, with a lifetime incidence of about 10% and a 5-year recurrence rate of 50% (1, 2). Furthermore, the prevalence of nephrolithiasis has been rising during the last decades, resulting in increased economic burden on the health care system (1, 3). The incidence of urolithiasis is related to geographical, climatic, ethnic, genetic and dietary factors. Urinary stone incidence depends on Urine becomes excessively supersaturated with urine mineral or certain relatively insoluble drugs, resulting in crystal formation, growth, aggregation and retention. Calcium oxalate (CaOx) is the main constituent in about 80% of kidney stones (4), many of which grow over depositions of calcium phosphate (CaP) called Randall plaques (RP), which are attached to the renal papillary surface. Infection stones are typically caused by infection with urease-producing bacteria. Hypercalciuria caused by Hyperparathyroidism and Cushing’s disease may cause kidney stone by boosting supersaturation of calcium oxalate or phosphate (5, 6). Obesity, hypertension, diabetes, and metabolic syndrome are also risk factors for stone formation (4). Nowadays, dietary habits are considered crucial factors in the formation of kidney stones (7), such as intakes of fluid, calcium, sodium and animal proteins. Urinary stone formation can be reduced by improving fluid intake. Excessive intake of animal protein, calcium and sodium should be avoided to prevent calcium stone formation. Excessive intake of oxalate may increase the incidence of calcium oxalate stones (8).

Selenium was considered a toxin until 1957 when Schwartz and Foltz discovered the protective effect of selenium on liver in rats and was later proved to be an essential trace element in human body by numerous experiments. Products with rich selenium content include fish, meat, offal, cereals and plants of the Brassica genus. Taking supplements containing organic selenium is a quick and effective way (9). Selenium reserves accumulate during the first half of our lives and the daily requirement mainly depends on age and state of our body. Combining selenium with vitamin E can strengthen the antioxidant protection and antagonize heavy metal toxicity. It is essential to identify the status and consumption of selenium for a specific community duo to the highly variable levels of selenium between diverse populations and regions (10).

Selenium has multiple and complex effects on human health (11). It has been well-researched that selenium deficiency primarily affects heart muscle, joints, nephropathy and neurological diseases (12). Excess of selenium may cause gastrointestinal upsets, infertility, hair loss, skin rash, nervous system disorders and so on (9). However, recent evidence suggests that high selenium intake through food or dietary supplements does not prevent cancer in humans and may even increase the risk of type 2 diabetes (13–15). The biological functions of dietary selenium are achieved by selenoproteins whose active center is selenocysteine, and the best known is the antioxidant glutathione peroxidase (GPX) (11). In recent years, the role of selenium preventing atherosclerosis and CVDs has attracted significant attention. Several findings have shown an association between kidney stones and CVDs (16), although the causation has not been definitively established. Moreover, some animal experiments have suggested negative relationships between selenium and CaOx stones (17–19).

Based on these findings, we supposed that dietary selenium intake might be associated with the risk of kidney stones in humans. This study aimed to confirm the hypothesis with participants from the 2007 to 2018 National Health and Nutrition Survey (NHANES).

## Materials and methods

### Study population

For the current analysis, the six consecutive 2-year cycles of NHANES 2007–2008, 2009–2010, 2011–2012, 2013–2014, 2015–2016, and 2017–2018 were collected, as the questions about kidney stones were responded by participants during these cycles. There were 59,842 participants aged 0–80 years in NHANES 2007–2018. The exclusion criteria were as follows: (a) participants who had not completed the kidney stones survey (*n* = 25,163) (all participants aged less than 20 and average age is 8.3); (b) unknown selenium (*n* = 3,963) (these participants did not receive 24-h dietary recall interview and we failed to obtain any dietary data); (c) pregnancy (*n* = 267); (d) unknown body mass index (BMI) (*n* = 318); (e) unknown diabetes (*n* = 15); (f) unknown hypertension (*n* = 39); (g) unknown recreational activities (*n* = 7); (h) unknown smoking data (*n* = 14); (i) abnormal selenium (*n* = 1501). And a total of 28,555 participants were included in the study.

### Study variables and outcome

The dietary intake data were obtained from a 24-h dietary recall interview with all participants. The intakes of selenium and other components from foods and beverages were calculated using the United States Department of Food and Nutrient Database for Dietary Studies (FNDDS). The first 24-h dietary recall interview (Dietary Interview - Total Nutrient Intakes, First Day) was used in the present study.

Based on the previous studies on dietary intake and kidney stones (20, 21), we included the following covariates: age (<50 years and ≥50 years), sex (male/female), race (Mexican American, other Hispanic, Non-Hispanic white, Non- Hispanic black and other), marital status (married and unmarried), education level (less than 11th grade, high school or equivalent, some college or AA degree, and college graduate or above), vigorous and moderate recreational activities, annual family income ($0–$19,999, $20,000–$44,999, $45,000–$74,999, ≥ $75,000 and other), hypertension, diabetes, BMI (< 25.0 kg/m^2^ and ≥ 25.0 kg/m^2^), smoking, daily intake of total energy, water, caffein, alcohol, calcium, phosphate, potassium, sodium, magnesium, and vitamins A, B6, C, D, E and K.

The NHANES 2007-2018 (Kidney Conditions – Urology) provides personal interview data on kidney stones for participants aged 20 years and older. And we considered participants who answered “Yes” to the question “Have you ever had kidney stones?” (KIQ026) as having a history of nephrolithiasis.

### Statistical analysis

Data were described as mean ± standard deviation (SD) for continuous variables and percentages (%) for categorical variables. The Kruskal Wallis test was used to evaluated continuous variables because of the non-normal distribution for dietary intake data, and chi-square (χ2) tests were used to analyze the categorical variables. We calculated quartiles based on the participants without kidney stone and calculated quartiles in each subgroup and analyzed to better match different populations. Three different multiple logistic regression models were used to calculate odds ratios (ORs) and 95% confidence interval (CI) for each quartile of selenium to kidney stones. Model 1 was the crude model, and we adjusted for age, sex and race in Model 2. And in Model 3, we further adjusted for the covariates of marital status, education level, vigorous and moderate recreational activities, annual family income, hypertension, diabetes, BMI, smoking, daily intake of total energy, water, caffein, alcohol, calcium, phosphate, potassium, sodium, magnesium, and vitamins A, B6, C, D, E, and K.

After an adjustment for potential confounders, we performed non-linear correlation by smoothing plot with three knots located at the 5th, 50th, and 95th percentiles of selenium intake based on the relevant study (22). If the non-linear correlation was founded, we further performed a two-piecewise linear regression model to calculate the threshold effect of the dietary selenium intake on kidney stones according to the smoothing plot. The inflection point was automatically calculated by recursive method when the relationship between the two became obvious in smoothed curve (23). All statistical analyses were performed using the software EmpowerStats.^1^ Two-tailed P values < 0.05 was considered as a statistically significant difference.

## Results

### Participant characteristics

**FIGURE 1:**
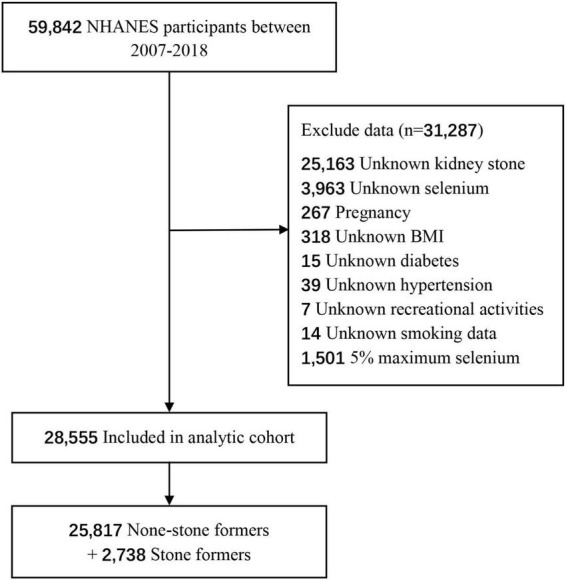
Flowchart of the participants from National Health and Nutrition Examination Survey (NHANES) in the present study.

We included 28,555 participants from NHANES 2007 to 2018 according to the inclusion and exclusion criteria, including 25,817 (90.4%) cases without kidney stones and 2,738 (9.6%) cases with kidney stones (Figure 1). Characteristics of participants are presented as two groups in Table 1. There are significant statistical differences among several variables, including age (*P* < 0.01), sex (*P* < 0.01), race (*P* < 0.01), marital status (*P* < 0.01), vigorous recreational activities (*P* < 0.01), moderate recreational activities (*P* < 0.01), education level (*P* < 0.01), hypertension (*P* < 0.01), diabetes (*P* < 0.01), BMI (*P* < 0.01), smoked at least 100 cigarettes in life (*P* < 0.01), total water drank (*P* < 0.01), daily intake of alcohol (*P* < 0.01), caffeine (*P* < 0.01), selenium (*P* = 0.02), phosphorus (*P* = 0.01), magnesium (*P* < 0.01), Vitamin B6 (*P* < 0.01) and Vitamin C (*P* < 0.01). Obviously, participants with kidney stones were more likely to be male (54.3%), aged more than 50 years (66.4%), non-Hispanic white (55.1%), married (57.2%), less vigorous recreational activities (85.3%), less moderate recreational activities (64.4%), some college or AA degree, hypertension-positive, diabetes-positive and BMI ≥ 25.0 kg/m^2^ (80.8%).

**TABLE 1:** Characteristics of participants in National Health and Nutrition Examination Survey (NHANES) 2007–2018.

Characteristic	None-stone No. (%)	Stone No. (%)	*P*-value
Total participants	25817 (90.41)	2738 (9.59)	
**Age**			<** 0.01**
Mean (SD)	49.43 (17.69)	56.44 (15.98)	
< 50 years	13059 (50.58)	919 (33.57)	
≥ 50 years	12758 (49.42)	1819 (66.44)	
**Sex**			<** 0.01**
Male	12020 (46.56)	1487 (54.31)	
Female	13797 (53.44)	1251 (45.69)	
**Race**			**< 0.01**
Mexican American	3900 (15.11)	338 (12.34)	
Other Hispanic	2660 (10.30)	321 (11.72)	
Non-Hispanic White	10492 (40.64)	1509 (55.11)	
Non-Hispanic Black	5765 (22.33)	361 (13.19)	
Other	3000 (11.62)	209 (7.63)	
**Marital status**			**< 0.01**
Married	13036 (50.49)	1567 (57.23)	
Unmarried	12781 (49.51)	1171 (42.77)	
Vigorous recreational activities			<** 0.01**
Yes	5680 (22.00)	402 (14.68)	
No	20137 (78.00)	2336 (85.32)	
**Moderate recreational activities**			<** 0.01**
Yes	10448 (40.47)	975 (35.61)	
No	15369 (59.53)	1763 (64.39)	
**Education**			<** 0.01**
Less than 11th grade	6249 (24.21)	691 (25.24)	
High school or equivalent	5938 (23.00)	617 (22.54)	
Some college or AA degree	7590 (29.40)	867 (31.67)	
College graduate or above	6040 (23.40)	563 (20.56)	
**Annual family income**			0.34
0–$19 999	6005 (23.62)	653 (24.19)	
20 000 to $44 999	8096 (31.84)	888 (32.90)	
45 000 to $74 999	4466 (17.56)	479 (17.75)	
≥$ 75 000	5939 (23.36)	593 (21.97)	
Other	921 (3.62)	86 (3.19)	
**Hypertension**			<** 0.01**
Yes	9151 (35.45)	1398 (51.06)	
No	16666 (64.55)	1340 (48.94)	
**Diabetes**			<** 0.01**
Yes	3205 (12.41)	616 (22.50)	
No/Borderline	22612 (87.59)	2122 (77.50)	
**BMI (kg/m^2^)**			<** 0.01**
Mean (SD)	29.17 (7.01)	30.57 (6.87)	
< 25.0	7552 (29.25)	527 (19.25)	
≥ 25.0	18265 (70.75)	2211 (80.75)	
**Smoked at least 100 cigarettes in life (%)**			<** 0.01**
Yes	11253 (43.59)	1382 (50.48)	
No	14564 (56.41)	1356 (49.53)	
**Daily intake [Mean (SD)]**			
Total energy (kcal)	2001.23 (858.47)	1969.50 (827.45)	0.15
Total water drank (g)	1088.02 (1172.16)	1035.39 (1171.61)	<** 0.01**
Alcohol (g)	9.77 (27.36)	6.93 (26.28)	<** 0.01**
Caffeine (mg)	146.93 (200.39)	168.77 (234.70)	<** 0.01**
Selenium (mcg)	103.02 (47.18)	100.44 (45.36)	**0.02**
Calcium (mg)	879.99 (530.57)	860.99 (517.00)	0.10
Phosphorus (mg)	1266.65 (572.53)	1238.11 (556.52)	**0.01**
Sodium (mg)	3261.34 (1570.66)	3227.70 (1519.60)	0.43
Potassium (mg)	2500.64 (1143.26)	2465.67 (1139.89)	0.08
Magnesium (mg)	282.93 (135.43)	272.33 (131.32)	<** 0.01**
Vitamin A (mcg)	580.94 (620.27)	583.20 (621.08)	0.26
Vitamin B6 (mg)	1.96 (1.55)	1.87 (1.31)	<** 0.01**
Vitamin C (mg)	81.67 (93.34)	75.42 (90.60)	<** 0.01**
Vitamin D (mcg)	4.22 (4.97)	4.18 (4.84)	0.51
Vitamin E (mg)	7.78 (5.87)	7.70 (5.84)	0.42
Vitamin K (mcg)	108.34 (192.91)	99.32 (136.27)	0.07

SD, standard deviation; BMI, body mass index. The bold values refer to P < 0.05, indicating significant statistical differences.

### Quartile analysis and non-linearity analysis

Multiple logistic regression models indicated that the fourth quantile (Q4) of dietary selenium intake had a lower risk of nephrolithiasis than the first quantile (Q1) in Model 1 (OR 0.87, 95% CI 0.78–0.97, *P* < 0.05), Model 2 (OR 0.85, 95% CI 0.75–0.95, *P* < 0.01) and Model 3 (OR 0.82, 95% CI 0.70–0.97, *P* < 0.05), while there were no statistical differences between the second quantile (Q2)/the third quantile (Q3) and Q1 (Table 2). The results showed that in stratified analysis by age, we found statistical differences between Q4 and Q1 for participants aged less than 50 in Model 1 (OR 0.75, 95% CI 0.62–0.91, *P* < 0.01), Model 2 (OR 0.73, 95% CI 0.60–0.89 *P* < 0.01) and Model 3 (OR 0.65, 95% CI 0.49–0.86, *P* < 0.01). But there was no positive correlation between selenium and kidney stones in cases aged more than 50. In stratified analysis by sex, there were statistical differences between Q4 and Q1 for male participants in Model 1 (OR 0.69, 95% CI 0.59–0.81, *P* < 0.01), Model 2 (OR 0.78, 95% CI 0.66–0.92 *P* < 0.01) and Model 3 (OR 0.73, 95% CI 0.58–0.91, *P* < 0.01). But we failed to find statistical significance for female participants. In stratified analysis by BMI, there were statistical differences between Q4 and Q1 for individuals with a BMI ≥ 25.0 in Model 1 (OR 0.88, 95% CI 0.77–0.99, *P* < 0.05), Model 2 (OR 0.84, 95% CI 0.73–0.95 *P* < 0.01) and Model 3 (OR 0.80, 95% CI 0.66–0.96, *P* < 0.05). But there were no statistical differences for individuals with a BMI < 25.0.

**TABLE 2:** Multivariate analysis of kidney stones by quartiles of selenium intake, National Health and Nutrition Examination Survey (NHANES) 2007–2018.

	Cutoff (mcg)	None-stone No. (%)	Stone No. (%)	Model 1 OR (95% CI)	Model 2 OR (95% CI)	Model 3 OR (95% CI)
**Overall**						
Q1	< 67.8	6432 (90.0)	718 (10.0)	1.00	1.00	1.00
Q2	67.8–97.2	6458 (90.2)	701 (9.8)	0.97 (0.87–1.09)	0.95 (0.85–1.06)	0.94(0.83–1.06)
Q3	97.2–133.8	6461 (90.3)	693 (9.7)	0.96 (0.86–1.07)	0.93 (0.83–1.05)	0.92 (0.80–1.05)
Q4	≥ 133.8	6466 (91.2)	626 (8.8)	0.87 (0.78–0.97)*	0.85 (0.75–0.95)**	0.82 (0.70–0.97)*
*P* value				0.01	< 0.01	0.02
**Age**						
**< 50 years**						
Q1	<72.1	3320 (92.5)	262 (7.5)	1.00	1.00	1.00
Q2	72.1–103.3	3269 (93.3)	234 (6.7)	0.89 (0.73–1.06)	0.87 (0.72–1.04)	0.83 (0.68–1.02)
Q3	103.3–141.2	3273 (93.7)	221 (6.3)	0.83 (0.69–1.00)	0.81 (0.67–0.98)	0.77 (0.62–0.97)
Q4	≥ 141.2	3297 (94.2)	202 (5.8)	0.75 (0.62–0.91)**	0.73 (0.60–0.89)**	0.65 (0.49–0.86)**
*P* trend				< 0.01	<0.01	< 0.01
**≥ 50 years**						
Q1	< 63.8	3201 (88.0)	436 (12.0)	1.00	1.00	1.00
Q2	63.8–91.6	3211 (88.0)	437 (12.0)	1.00 (0.87–1.15)	0.93 (0.81–1.08)	0.93 (0.80–1.09)
Q3	91.6–125.5	3148 (86.5)	491 (13.5)	1.15 (1.00–1.32)	1.02 (0.89–1.18)	1.02 (0.86–1.20)
Q4	≥ 125.5	3198 (87.5)	455 (12.5)	1.05 (0.91–1.20)	0.87 (0.75–1.01)	0.86 (0.70–1.05)
*P* trend				0.31	0.12	0.22
**Gender**						
**Male**						
Q1	< 79.5	2961 (88.0)	404 (12.0)	1.00	1.00	1.00
Q2	79.5–112.4	2961 (87.5)	423 (12.5)	1.05 (0.91–1.21)	1.06 (0.91–1.23)	1.02 (0.87–1.20)
Q3	112.4–149.8	3009 (89.1)	369 (10.9)	0.90 (0.77–1.04)	0.94 (0.81–1.09)	0.90 (0.75–1.08)
Q4	≥ 149.8	3089 (91.4)	291 (8.6)	0.69 (0.59–0.81)**	0.78 (0.66–0.92)**	0.73 (0.58–0.91)**
*P* trend				< 0.01	<0.01	< 0.01
**Female**						
Q1	< 60	3410 (90.8)	347 (9.2)	1.00	1.00	1.00
Q2	60.1–85.4	3455 (92.0)	301 (8.0)	0.86 (0.73–1.01)	0.86 (0.73–1.02)	0.84 (0.71–1.00)*
Q3	85.5–116.5	3462 (91.9)	304 (8.1)	0.86 (0.74–1.01)	0.89 (0.76–1.05)	0.85 (0.71–1.03)
Q4	≥ 116.5	3470 (92.1)	299 (7.9)	0.85 (0.72–1.00)*	0.93 (0.79–1.09)	0.86 (0.68–1.09)
*P* trend				0.07	0.52	0.30
**BMI (kg/m^2^)**						
**< 25.0**						
Q1	<66.9	1880 (93.1)	140 (6.9)	1.00	1.00	1.00
Q2	66.9–96.7	1871 (92.8)	145 (7.2)	1.04 (0.82–1.32)	1.04 (0.81–1.33)	1.09 (0.84–1.42)
Q3	96.7–133.8	1892 (93.7)	127 (6.3)	0.90 (0.70–1.16)	0.95 (0.74–1.23)	0.98 (0.72–1.32)
Q4	≥ 133.8	1909 (94.3)	115 (5.7)	0.81 (0.63–1.04)	0.88 (0.67–1.15)	0.95 (0.65–1.38)
*P* trend						
**≥ 25.0**						
Q1	< 67.9	4533 (88.6)	582 (11.4)	1.00	1.00	1.00
Q2	67.9–97.0	4570 (89.3)	548 (10.7)	0.93 (0.83–1.06)	0.91 (0.80–1.03)	0.89 (0.78–1.02)
Q3	97.0–133.1	4550 (89.0)	562 (11.0)	0.96 (0.85–1.09)	0.92 (0.81–1.04)	0.90 (0.78–1.05)
Q4	≥ 133.1	4612 (89.9)	519 (10.1)	0.88 (0.77–0.99)*	0.84 (0.73–0.95)**	0.80 (0.66–0.96)*
*P* trend				0.06	0.01	0.03

Model 1: no covariates were adjusted.

Model 2: adjusted for gender, age and race.

Model 3: adjusted for gender, age, race, marital status, vigorous and moderate recreational physical activity, education level, annual family income, hypertension, diabetes, BMI (body mass index), smoking, energy, water, dietary intakes of alcohol, caffeine, calcium, phosphate, sodium, potassium, magnesium, vitamins A, C, D, E, K.

*P < 0.05, **P < 0.01.

**FIGURE 2:**
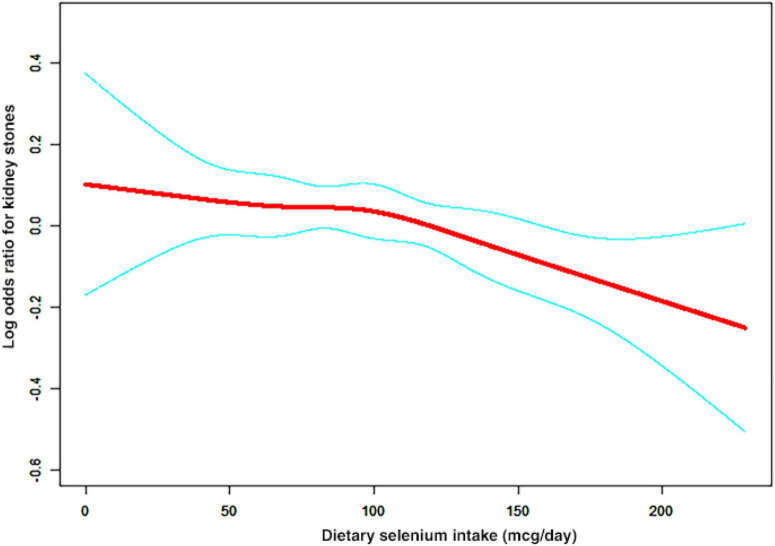
The association curve between dietary selenium intakes and the risk of kidney stones. The solid red line represents the smooth curve fit between variables and the blue line represents the 95% of confidence interval from the fit.

**FIGURE 3:**
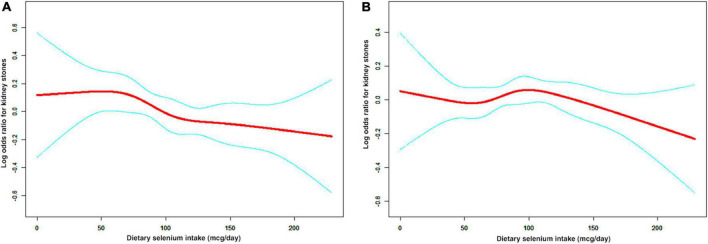
The non-linear relationship between dietary selenium intakes and the risk of kidney stones in stratified analysis by age. **(A)** age < 50 years group, **(B)** age ≥ 50 years group.

**FIGURE 4:**
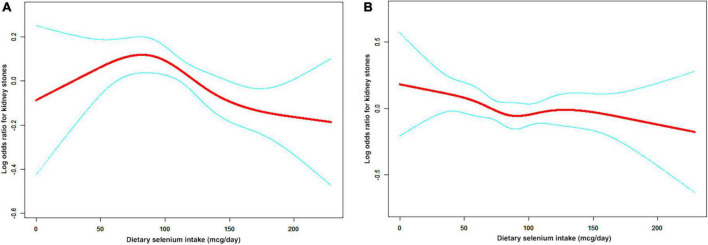
The non-linear relationship between dietary selenium intakes and the risk of kidney stones in stratified analysis by sex. **(A)** male group, **(B)** female group.

**FIGURE 5:**
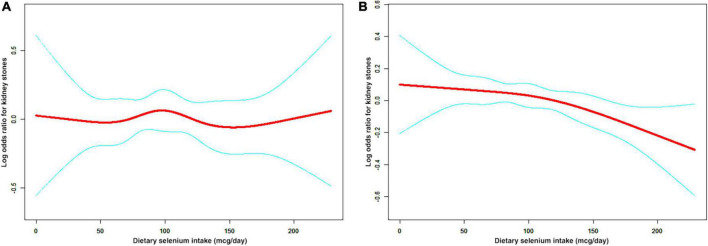
The non-linear relationship between dietary selenium intakes and the risk of kidney stones in stratified analysis by BMI. **(A)** BMI < 25.0 group **(B)** BMI ≥ 25.0 group.

A non-linear relationship between dietary selenium intake and kidney stones was founded in all participants (Figure 2), younger (Figure 3A), male (Figure 4A) and overweight/obese (Figure 5B) individuals after adjusting for confounding factors. But the non-linear relationship can not be founded in older (Figure 3B), female (Figure 4B) and people with a BMI < 25.0 (Figure 5A). The two-piecewise linear regression model was used to calculate threshold effect and we founded the inflection points were 99.2, 36.3, 57.8, and 47.5 (mcg/day) in all participants, younger, male and overweight/obese individuals, respectively, after an adjustment for potential confounders (Table 3).

**TABLE 3:** Threshold effect analysis of dietary selenium intake on the prevalence of kidney stones using piece-wise linear regression.

Inflection points (mcg/day)	Adjusted* OR (95% CI)	*P*-value
**All participants**		
<99.2	1.000 (0.997, 1.002)	0.747
≥ 99.2	0.998 (0.996, 0.999)	**0.012**
**Age**		
<50 years		
<36.3	1.014 (0.992, 1.035)	0.216
≥36.3	0.998 (0.995, 0.999)	**0.041**
≥50 years		
<114.8	1.001 (0.999, 1.003)	0.428
≥114.8	0.997 (0.995, 1.000)	0.053
**Gender**		
Male		
<57.8	1.006 (0.998, 1.015)	0.149
≥57.8	0.998 (0.996, 0.999)	**0.010**
Female		
<69	0.996 (0.991, 1.001)	0.110
≥69	1.000 (0.997, 1.002)	0.716
**BMI (kg/m^2^)**		
<25.0		
<83.1	1.001 (0.995, 1.007)	0.763
≥83.1	0.999 (0.995, 1.002)	0.539
≥25.0		
<47.5	1.004 (0.996, 1.012)	0.368
≥47.5	0.998 (0.997, 0.999)	**0.010**

*Adjusted for gender, age, race, marital status, vigorous and moderate recreational physical activity, education level, annual family income, hypertension, diabetes, BMI (body mass index), smoking, energy, water, dietary intakes of alcohol, caffeine, calcium, phosphate, sodium, potassium, magnesium, vitamins A, C, D, E, K. The bold values refer to P < 0.05, indicating significant statistical differences.

## Discussion

We confirmed dietary selenium intake was inversely associated with the incidence of kidney stones in adults from the 2007 to 2018 NHANES, which is the first study to discovery that dietary selenium can prevent kidney stones in humans. Furthermore, the stratified analyses indicated there were statistical differences between dietary selenium intake and kidney stones among younger (age < 50), male and overweight/obese (BMI ≥ 25.0) individuals.

With the development of modern endoscopy techniques, the correlation between RP and CaOx stones has been well documented. According to the vascular theory, renal papillary circulation is apt to injury because of turbulent flow, relative hypoxia, and hyperosmolarity environment, which increases the likelihood of atherosclerotic-like reaction and precipitates plaque formation during the repair (24). Experimental studies showed that exposure to high oxalate, CaOx or CaP crystals can induce inflammatory response and biomineralization in renal cells and lots of crystallization modulators and inhibitors participant in crystal nucleation, growth, aggregation and retention. Then nearby cells react to the foreign body by producing reactive oxidative stress (ROS) (25). Once the generation of ROS is uncontrolled or the endogenous antioxidant capacity is decreased, oxidative stress (OS) will be created and may result in inflammation and injury, which increases intracellular levels of antioxidants (25).

Previous studies revealed that RP were a process of biomineralization, sharing similarities with atherosclerotic plaques (24, 26). Plenty of clinical and laboratory studies suggests that OS is also an important feature in the pathogenesis of atherosclerosis. Risk factors for atherosclerosis such as hypertension, diabetes and hypercholesterolemia increase the production of ROS in the arterial wall, leading to oxidative stress (27). Some experiments *in vitro* and vivo support the anti-atherosclerosis effect of selenium mainly owing to its antioxidant properties. As the biologically active form of selenium, selenoproteins can prevent atherosclerosis and CVDs by inhibiting OS, inflammation reaction, endothelial dysfunction and vascular calcification (27). And a meta-analysis of 25 studies showed a significant inverse association between blood selenium status and coronary heart disease risk (28).

It is reasonable to speculate that selenium may inhibit the formation of kidney stones through a similar pathway. In addition, the protective effect of selenium is more evident in young individuals, because the incidence of CVDs in elderly patients is significantly increased, indicating that the ability to resist ectopic calcification is greatly weakened, and the protective effect of selenium on kidney stones is covered up.

Only three animal experiments so far have suggested inverse associations between selenium and CaOx stones. Sakly et al. reported for the first time that intraperitoneal injection of selenium greatly inhibited CaOx deposition in rats (18). Another study indicated that supplementation of vitamin E and selenium reduced the level of renal lipid peroxidation and the activities of oxalate biosynthetic enzymes in rats with feeding calculi producing diet (17). And the lipid peroxide production was associated with renal cell damage that was caused by oxalate and CaOx crystals (29). A recent study in dogs further suggested that hyperoxaluria caused the excessive osteopontin (OPN) expression, and dietary selenium may inhibit CaOx stones by downregulating OPN expression (19). The previous studies concluded that OPN is able to inhibit the formation and retention of CaOx crystal in the kidney *in vivo* (30).

We discovered a relation between kidney stones and dietary selenium intake in men, but not in women. A potential explanation is that sex greatly influences the metabolism of ROS in the body. Several studies found that estrogen and estrogen receptor signaling pathways might suppress oxidative stress-induced renal cell injury (31, 32). Zhu et al. recently reported estrogen receptor β signals may inhibit renal CaOx crystal deposition by reducing OS in tubular cells (33). Compared with males, females mitochondria produced significantly less hydrogen peroxide and higher amounts of GPX, reduced glutathione and manganese superoxide dismutase (34). Therefore, the complex anti-oxidative mechanisms in female might explain our failure to discover any association between kidney stones and dietary selenium. Contrary to estrogen, androgen and androgen receptor signaling might influence the anabolism of ROS, leading to oxidative stress-induced renal injury and further renal CaOx crystal deposition (35). But selenoproteins can prevent renal CaOx deposition in males by inhibiting OS. Selenium has been known to support the production of testosterone in laboratory and animal models (36), not in humans. And which mechanism dominates remains to be proved.

Furthermore, some animal studies indicated sexual differences in selenium distribution and selenoprotein expression in various organs are distinctly different, which vary with selenium status and individual age (37, 38). Selenoprotein P (SeP) participates in the storage and transport of about 60% of plasma selenium (39). Lutz et al discovered that renal SeP mRNA concentrations were 1.7-fold higher in male mice than in female mice, and the difference is sustained with age. Meanwhile, renal mRNA concentrations of Glutathione peroxidase 1 (GPX1) and Glutathione peroxidase 3 (GPX3) displayed no significant sex differences in both young and old mice (37).

We also discovered that dietary selenium intake was inversely associated with kidney stones in overweight/obese individuals. Numerous findings have confirmed that BMI is related to the incidence of urolithiasis in the last decade (40–42). The accumulation of visceral fat is a risk factor for nephrolithiasis (43), and mounting evidence links adipose cells to urinary stone formation (44, 45). Fatty acid binding protein 4 (*FABP4*), mainly expressed in adipocytes and macrophages, is involved in lipid transfer and transport and significantly correlates with plasma lipid levels (46). A recent study demonstrated that lipid metabolism in renal papillary tissue containing RP was impaired, which was associated with the downregulation of *FABP4* based on immunohistochemistry of human renal tissue, microarray analysis of nephrocalcinosis model mice and *FABP4* knockout mice (47).

Previous studies observed a positive correlation between plasma selenium levels and total, TG and LDL cholesterol (46, 48), although there were inconsistent for HDL cholesterol in some studies (49). Galan-Chilet et al. found statistical interactions of selenium status with genetic variation (such as *FABP4*) in lipid metabolic pathways, indicating potentially interconnected pathways in selenium and lipid transport and transfer (46). Another study showed a positive association between *FABP4* and plasma selenium levels and a negative association between *FABP4* and GPX3 activity in Indonesian men with visceral obesity, suggesting selenium status may play different roles in obese people, such as in OS condition and inflammatory process (50).

To our knowledge, this is the first population-based study to examine the association between dietary selenium intake and the risk of kidney stones. Using data from large and consecutive nationally representative surveys, we can assess the associations between selenium and kidney stones by both quartile analysis and dose-response analysis after adjusting potential confounding variables. However, the limitations of the study deserve mention. Firstly, causality cannot be proved because of the cross-sectional survey study. Secondly, selection bias cannot be completely avoided because the formation of kidney stones is affected by numerous factors. Thirdly, a person’s long-term intake habits may not be set in stone, and cannot be accurately described by a single 24-h dietary recall, meaning that kidney stones may have occurred before participants changed their dietary habits. However, data from NHANES has been used for decades in epidemiological studies and health sciences research. Fourthly, our findings are only representative of the United States population. Because human dietary selenium intake varies by country, depending on soil and geography (12). Fifthly, no information is available on stone composition that may further illuminate the relationship between selenium and kidney stones. Sixthly, we can’t know whether dietary selenium intakes can prevent phosphate stones, urate stones and cystine stones, relieve renal colic, or affect the recurrence rate of kidney stones in children and in heredity. Finally, despite the biological plausibility, additional longitudinal and laboratory studies are needed to confirm our results and elucidate the potential mechanisms.

## Conclusion

Our study revealed an inverse relation between the level of dietary selenium intake and the risk of kidney stones for the United States population, especially for younger (age < 50), male and overweight/obese (BMI ≥ 25.0) individuals. Increasing dietary selenium intake might be meaningful to the prevention of kidney stones. Further studies are required to confirm our findings and clarified the biological mechanisms.

## Data availability statement

Publicly available datasets were analyzed in this study. This data can be found here: https://www.cdc.gov/nchs/nhanes/index.htm.

## Ethics statement

The studies involving human participants were reviewed and approved by Institutional Review Board of the National Center for Health Statistics (NCHS). The patients/participants provided their written informed consent to participate in this study.

## Author contributions

ML: project development, data analysis, and manuscript writing. ZC, JC, and MG: data collection and analysis. ZZ and HC: project development and manuscript editing. All authors contributed to the article and approved the submitted version.

## Conflict of interest

The authors declare that the research was conducted in the absence of any commercial or financial relationships that could be construed as a potential conflict of interest.

## Publisher’s note

All claims expressed in this article are solely those of the authors and do not necessarily represent those of their affiliated organizations, or those of the publisher, the editors and the reviewers. Any product that may be evaluated in this article, or claim that may be made by its manufacturer, is not guaranteed or endorsed by the publisher.

## Footnotes

1
http://www.empowerstats.com

## Abbreviations

CaOx: Calcium oxalate
CaP: calcium phosphate
RP: Randall plaques
CVDs: cardiovascular diseases
GPX: glutathione peroxidase
NHANES: National Health and Nutrition Survey
BMI: body mass index
Q1: the first quantile
Q2: the second quantile
Q3: the third quantile
Q4: the fourth quantile
OS: oxidative stress
OPN: osteopontin
ROS: reactive oxygen species
SeP: Selenoprotein P
GPX1: Glutathione peroxidase 1
GPX3: Glutathione peroxidase 3
*FABP4*: Fatty acid binding protein 4.
